# Genome-wide identification of the *DFR* gene family in *Dracaena cambodiana* and its expression analysis under wound stress

**DOI:** 10.1186/s12870-026-08486-x

**Published:** 2026-03-06

**Authors:** Shuang Li, Hongyou Zhao, Chunyong Yang, Yanfang Wang, Yating Zhu, Qianxia Li, Ge Li, Lixia Zhang, Zhaoyou Deng, Ling Wang, Yanqian Wang

**Affiliations:** 1 Yunnan Key Laboratory of Southern Medicine Utilization, Yunnan Branch of Institute of Medicinal Plant Development, Chinese Academy of Medical Sciences & Peking Union Medical College, Jinghong, 666100 China; 2 Institute of Medicinal Plant Development, Chinese Academy of Medical Sciences & Peking Union Medical College, Beijing, 100193 China; 3 Yunnan Key Laboratory of Sustainable Utilization of Southern Medicine, Yunnan University of Traditional Chinese Medicine, Kunming, 650500 China

**Keywords:** Dihydroflavonol 4-reductase, Tandemly duplicated gene clusters, Flavonoid, Dragon's blood

## Abstract

*Dracaena cambodiana* Pierre ex Gagnep. is divided into two distinct groups: the Yunnan clade with soft leaves (*D. cambodiana* A) and the Hainan clade with hard leaves (*D. cambodiana* B). This species is the key plant source of dragon’s blood, a well-known traditional Chinese medicine derived from the defensive metabolites of *Dracaena* species, with flavonoids as its major bioactive components. Dihydroflavonol 4-reductase (DFR) plays a pivotal role in flavonoid biosynthetic pathway. In this study, we identified 19 *DFR* genes (designated as *DcDFR*s) from the reference genome of *D. cambodiana*, which are distributed across six chromosomes and phylogenetically classified into four subfamilies. Comprehensive analyses of their chromosomal localization, sequence homology, gene structure, and phylogenetic relationships revealed two distinct tandemly duplicated gene clusters (TDGCs), designated as TDGCs-1 and TDGCs-2. Notably, TDGCs-1, comprising *DcDFR1*–*DcDFR5* and localized on chromosome 5, exhibits conserved tissue-specific co-expression patterns and coordinated transcriptional responses to wound stress across two distinct *D. cambodiana* accessions (*D. cambodiana* A and *D. cambodiana* B). Its expression levels are positively correlated with both the duration of stress induction and flavonoid accumulation, suggesting a critical role in flavonoid metabolism and wound defense responses. Unlike TDGCs-1, TDGCs-2 does not show typical co-expression patterns under wound stress in *D. cambodiana* A, which implies that the two tandemly duplicated gene clusters have both overlapping and distinct biological functions. This study reveals the secondary metabolic pathways triggered by particular environmental stresses and provides a theoretical foundation for future investigations into the artificial induction technology of dragon’s blood.

**Supplementary Information:**

The online version contains supplementary material available at 10.1186/s12870-026-08486-x.

## Introduction

*Dracaena cambodiana* Pierre ex Gagnep., an ancient monocotyledonous evergreen tree within the genus *Dracaena*, has two distinct groups: the Yunnan clade with soft leaves (*D. cambodiana* A) and Hainan clade with hard leaves (*D. cambodiana* B) [1]. The tree species is mainly distributed in Yunnan, Guangxi, and Hainan Provinces in China, as well as in several Southeast Asian countries [2]. When the trunk of *D. cambodiana* is damaged by external factors, a red resin-like substance is secreted at the wound site. This substance is called dragon’s blood (also known as “XueJie”) [3] and is of great significance. Dragon’s blood is not only used as a traditional Chinese medicine but it also has extensive international applications. Consequently, *D. cambodiana* has emerged as an important medicinal plant for the production of dragon’s blood resin. Dragon’s blood has a variety of pharmacological properties, including enhancing blood circulation to dissipate blood stasis, alleviating inflammation and pain, and has the effect of astringing to arrest bleeding, which is used to treat wounds, leucorrhea, fractures, diarrhea and piles, as well as intestinal and stomach ulcers [4]. With the constant advancements in clinical medicine research, dragon’s blood has demonstrated promising efficacy in anti-cancer treatment [5, 6, 7] and the management of cardiovascular diseases [8, 9], and is being explored for its potential as an antidepressant [10, 11, 12]. In the modern industrial realm, owing to its unique color and chemical characteristics, Dragon’s blood is crucial in industries, such as cosmetic and coating industries [13, 14]. Nevertheless, *D. cambodiana* has a low growth rate and dragon’s blood yield in the natural environment is extremely low, which is not sufficient to meet the ever-increasing global demand. This has led to the overexploitation and destructive harvesting of wild *Dracaena* resources. In light of this, *D. cambodiana* and *D. cochinchinensis*, which are two key plant sources of dragon’s blood, have been enlisted in the “List of Wild Plants under State Key Protection (2021 Edition)” as second-class protected species [15]. Illegal digging, collection, and trading of these plants are explicitly prohibited.

Dragon’s blood is regarded as a defensive metabolite generated by *Dracaena* trees to counteract biotic and abiotic stresses. Dragon’s blood synthesis is a complex process that encompasses multiple enzymatic reactions and metabolic stages. Modern chemical and pharmacological investigations have demonstrated that the core active components of dragon’s blood comprise flavonoids, terpenoids, and other compounds [16]. Notably, the flavonoid content of dragon’s blood can reach 50–80% [17, 18]. The flavonoid components in dragon’s blood, including the active ingredients loureirin A and B, exhibit a C6-C3-C6 flavonoid skeleton structure that is analogous to that of anthocyanins and other flavonoids [19, 20]. The structure is derived from phenylalanine and malonyl coenzyme A. Chemically, they likely share certain precursor substances and intermediate metabolic steps during biosynthesis.

Dihydroflavonol 4-reductase (DFR), which is encoded by the *DFR* gene, is a crucial enzyme involved in the metabolism of flavonoids and polyphenols in plants [21, 22] and is essential for the synthesis of plant secondary metabolites. In a wide array of plants, *DFR* is intricately involved in the biosynthesis of anthocyanins and proanthocyanidins, where it catalyzes the reduction of dihydroflavonols to leucoanthocyanidins. Notably, *DFR* expression level is strongly correlated with the anthocyanin content. For example, anthocyanin accumulation in plants such as *Lycium ruthenicum Murr*. [23], *Hosta ventricosa [*24], *Paeonia suffruticosa *[25], *Brassica oleracea* var. *acephala *[26, 27], is enhanced when the *DFR* gene is highly expressed, which directly impacts the color of fruits, flowers, and leaves. However, *DFRs* derived from different plants exhibit different preferences for substrates (dihydrokaempferol [DHK]), dihydroquercetin [DHQ], dihydromyricetin [DHM]), which have been found in plants such as *Camellia sinensis *[28], *Ginkgo biloba *[29], *Medicago truncatula *[30], *Rhododendron delavayi *[31], and *Dryopteris erythrosora *[32]. Moreover, the engineered DFR gene in petunia can have a preference for specific substrates, thereby influencing​ anthocyanin synthesis and flower color [33, 34]. The *DFR* gene exhibits differential expression across various plant tissues and developmental stages. During fruit coloring of strawberry [35] and plum [36, 37], the expression levels of most *DFR* genes peak at the full fruit coloring stage. The *DFR* gene is also implicated in plant growth and development. Overexpression of the *GbDFR6* gene in *Ginkgo biloba* affects the growth and development of tobacco plants, leading to alterations in root and leaf morphology, as well as delayed flowering [38]. The expression level of the *DFR* gene changes dynamically in plants in response to biotic or abiotic stresses, such as bacterial leaf blight stress [39], salt stress [40, 41], drought stress [42], high temperature [43, 44] and sunburn stress [45], the expression level of the *DFR*, suggesting that it influences plant stress response. Studies have revealed that the regulation of tea [46], jujube [47], and grape [48] flavors is influenced by the *DFR* gene. Although the *DFR* gene is involved in numerous secondary metabolic reactions in plants, it exhibits sequence conservation in catalytic mechanisms and basic functions. This conservation not only provides a foundation for studying the functions of *DFR* genes in different plants but also implies its important position within the regulatory network of plant secondary metabolism.

Wang et al. [49] induced the production of dragon’s blood in *D. cochinchinensis* using *Fusarium proliferatum* and the chemical components of the induced dragon’s blood are almost the same as those of the natural dragon’s blood. Zhu et al. [50] artificially induced dragon’s blood production in *D. cambodiana* by injecting a chemical inducer and analyzed the transcriptome data. The researchers discovered that among the 16 genes annotated as *DFR* genes, the expression levels of seven *DFR* genes were considerably upregulated following inducer injection. The upregulation of gene expression was correlated with flavonoid accumulation. Six *DFR* genes in *D. cochinchinensis *[51] exposed to wound stress have been shown to be involved in the flavonoid biosynthetic pathway. The expression levels of five of these genes increase substantially after wound stress, which is consistent with the flavonoid accumulation. According to Yang et al., high expression levels of two *DFR* genes have been detected after six months of wound induction in *D. cochinchinensis *[52]. Genomic data analysis of *D. cochinchinensis* has revealed that *DFR* genes are enriched in the flavonoid biosynthesis and metabolic pathways [53]. Generally, a tandemly duplicated gene cluster (TDGCs) in plants contains three or more homologous genes, while a biosynthetic gene cluster (BGCs) contains three or more nonhomologous enzyme genes associated with the same secondary metabolic pathway [54]. This evidence suggests that the *DFR* gene is involved in flavonoid biosynthesis in dragon’s blood by catalyzing specific reaction steps, thereby converting precursor substances into the effective components present in dragon’s blood resin.

The identification and functional characterization of *DFR* genes in this plant species are associated with substantial knowledge gaps due to the scarcity of genomic resources of *D. cambodiana*. These gaps impeded our capacity to regulate the synthesis of dragon’s blood and boost its production via biotechnological approaches. Comprehending the evolutionary diversity and expression dynamics of *DFR* genes in *Dracaena* plants is crucial for enhancing our understanding of the biosynthesis of medicinally valuable flavonoids and the adaptive responses of the plant to environmental challenges. Therefore, this study aimed to address the gaps by conducting a systematic analysis of the *DFR* gene family in *D. cambodiana*. Leveraging the published genomic data and bioinformatics techniques, we identified 19 *DFR* homologous genes, which are distributed on six chromosomes, including two tandem duplication gene clusters that characterized their structural features, and evaluated their expression patterns under normal and wound stress conditions. The findings of this study will enhance our understanding of flavonoid biosynthesis in plant species of the genus *Dracaena*, thereby forming a theoretical foundation for elucidating the mechanisms by which *DFR* genes regulate dragon’s blood synthesis, as well as offer novel perspectives for increasing dragon’s blood production and improving the stress tolerance capacity of these endangered plants through human intervention.

## Results

### Identification and subcellular localization of DcDFR proteins

**Fig. 1:**
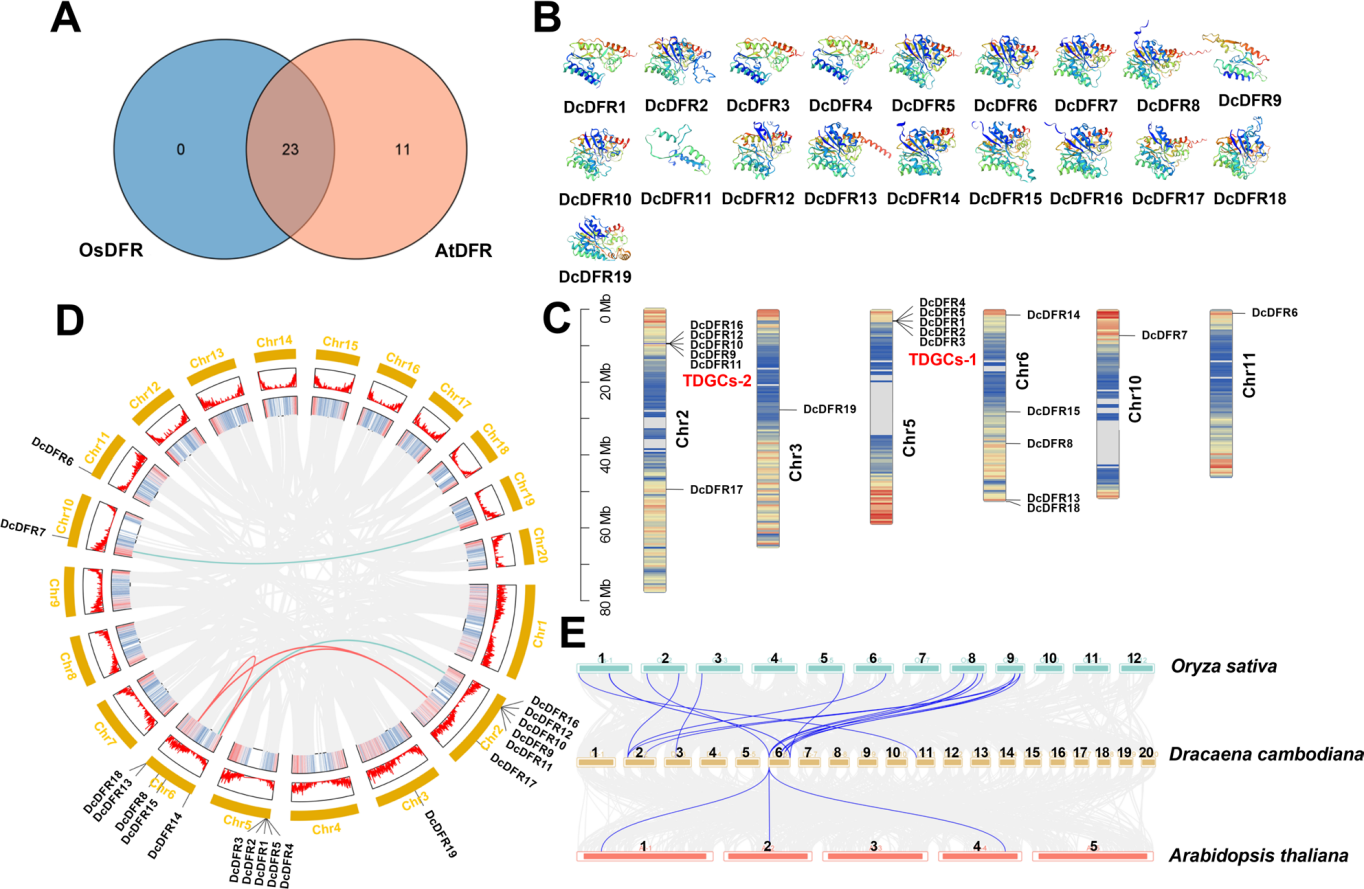
Evolutionary and structural characterization of *DcDFR* genes in *D. cambodiana. ***A** Venn diagram illustrating BLASTP results of OsDFR and AtDFR. **B** Three-dimensional (3D) structure models of DcDFR proteins. **C** Distribution of *DcDFR* genes on *D. cambodiana* chromosome. TDGCs, tandemly duplicated gene clusters. **D** Intraspecies collinearity: the red line represents collinear gene pairs of *DcDFR* genes, while the green line represents collinear gene pairs between *DcDFR* and undetected genes. **E** Interspecies collinearity: the blue line represents collinear relationships between *D. cambodiana* and *Oryza sativa*, and between *D. cambodiana* and *Arabidopsis thaliana*

To elucidate the evolution of the *DFR* family in *D. cambodiana*, a BLAST analysis was conducted using the DFR protein sequence from *Oryza sativa* and *Arabidopsis thaliana* (Fig. 1A). A total of 19 *DcDFR* genes were identified and named *DcDFR1*-*DcDFR19* after removing redundant and incomplete sequences (Table S1). Further amino acid sequence alignment revealed that the NADP-binding domain [31] was present in all DcDFRs except DcDFR1, DcDFR3, DcDFR4, DcDFR9, and DcDFR11 (Figure S1). Analysis of the physicochemical properties of DcDFR proteins (Table 1) revealed that the lengths of amino acids (Len) ranged between 208 and 545, molecular weights (MW) ranged from 22.52 to 61.25 kD, and the theoretical isoelectric points (pI) ranged from 5.21 to 8.55. All DcDFR proteins were stable except DcDFR1, DcDFR2, DcDFR3, DcDFR4, and DcDFR5. The aliphatic index (AI) values and grand average of hydrophobicity (GR) values ranged from 75.75 to 103.37, -0.282 to 0.13, respectively. The subcellular localization (SL) results revealed that DcDFR proteins were distributed in the chloroplast, cytoplasm, and nucleus. Among the 19 DFR proteins, DcDFR18 was localized in the nucleus, while DcDFR13 and DcDFR15 were predicted to be secreted proteins.

**Table 1:** Physicochemical properties of DcDFR proteins

Gene	Len (aa)	MW (kD)	pI	II	AI	GR	SL
*DcDFR1*	243	27.62	8.55	60.89	83.09	-0.062	Chloroplast
*DcDFR2*	365	40.93	6.86	49.8	79.32	-0.159	Chloroplast
*DcDFR3*	239	26.91	7.03	52.82	81.59	-0.054	Chloroplast
*DcDFR4*	240	27.13	6.66	47.23	82.46	-0.028	Chloroplast
*DcDFR5*	335	37.64	7.99	43.53	80.3	-0.127	Chloroplast
*DcDFR6*	320	35.62	5.74	37.83	84.38	-0.224	Cytoplasm
*DcDFR7*	326	35.38	5.76	37.59	89.82	-0.12	Chloroplast
*DcDFR8*	337	36.98	6.02	29.47	88.78	-0.151	Chloroplast
*DcDFR9*	252	27.46	5.26	37.7	103.37	0.269	Cytoplasm
*DcDFR10*	313	34.81	6.61	29.94	91.53	-0.103	Chloroplast
*DcDFR11*	208	22.52	5.21	33.66	94.71	0.13	Chloroplast
*DcDFR12*	545	61.25	5.99	32.41	91.71	-0.211	Cytoplasm
*DcDFR13*	352	39.25	5.89	36.94	90.54	-0.115	Secreted
*DcDFR14*	326	35.77	6.4	23.67	86.41	-0.136	Chloroplast
*DcDFR15*	335	37.30	6.16	33.1	94.87	0.004	Secreted
*DcDFR16*	322	35.98	5.7	31.93	91.4	-0.18	Cytoplasm
*DcDFR17*	293	32.12	8.3	31.14	91.77	-0.039	Chloroplast
*DcDFR18*	402	44.19	6.19	38.55	75.75	-0.282	Nucleus
*DcDFR19*	339	37.54	7.64	36.89	93.78	0.029	Chloroplast

*Len* Length of amino acids, *MW* Molecular weight, *pI* theoretical isoelectric points, *II* Instability index, *AI* Aliphatic index, *GR* Grand average of hydrophobicity, *SL* Subcellular localization

### Secondary structure and three-dimensional structure models of the *DcDFR* gene family

Analysis of the secondary structure proportion of the *DFR* gene family in *D. cambodiana* (Table 2) revealed that 19 family members were predominantly composed of alpha helices, accounting for 36.18–48.02% and random coils, which ranged from 33.04 to 46.44%. DcDFR9 had the highest percentage of alpha helices at 48.02% and DcDFR3 had the highest percentage of random coils at 46.44%. The proportion of extended strands ranged from 8.65% (DcDFR11) to 15.62% (DcDFR6) and that of beta turns ranged from 2.40% (DcDFR11) to 8.55% (DcDFR19). The SWISS-MODEL software was employed to predict the three-dimensional (3D) structure models of the 19 DcDFR proteins. According to the predicted three-dimensional structural model of the DFR proteins, the structural patterns of most family members exhibited considerable similarities (Fig. 1B). The similarity of the advanced structures of proteins suggests that they have the same functions.

**Table 2:** Secondary structure of DcDFR proteins

Gene	Alpha Helix	Extended Strand	Beta Turn	Random Coil
DcDFR1	39.51%	12.35%	4.53%	43.62%
DcDFR2	38.63%	13.97%	6.30%	41.10%
DcDFR3	38.08%	10.88%	4.60%	46.44%
DcDFR4	41.32%	11.16%	4.55%	42.98%
DcDFR5	41.79%	13.73%	6.57%	37.91%
DcDFR6	40.94%	15.62%	6.56%	36.88%
DcDFR7	40.49%	15.03%	6.44%	38.04%
DcDFR8	40.36%	13.95%	5.64%	40.06%
DcDFR9	48.02%	12.30%	3.97%	35.71%
DcDFR10	41.85%	15.34%	6.07%	36.74%
DcDFR11	46.15%	8.65%	2.40%	42.79%
DcDFR12	43.67%	13.03%	6.06%	37.25%
DcDFR13	40.34%	13.35%	6.53%	39.77%
DcDFR14	40.49%	14.42%	5.83%	39.26%
DcDFR15	42.09%	14.33%	5.37%	38.21%
DcDFR16	40.99%	14.60%	5.90%	38.51%
DcDFR17	36.18%	13.99%	7.17%	42.66%
DcDFR18	43.78%	10.45%	4.48%	41.29%
DcDFR19	44.25%	14.16%	8.55%	33.04%

### Chromosome localization and collinearity of *DcDFR* gene family

Based on the chromosome annotation data of *D. cambodiana*, a total of 19 *DcDFR* genes were unevenly dispersed across six chromosomes (Fig. 1C). Chromosome 2 had the highest number of *DcDFR*s genes (six). Both chromosomes 5 and 6 contained five *DcDFR* genes each. Conversely, chromosomes 3, 10, and 11 consisted of a solitary *DcDFR* gene. Notably, there were five *DFR* genes on both chromosomes 5 (*DcDFR1*–*5*) and 2 (*DcDFR9*–*12*, *16*) that were located within the same region. The distance between these genes ranged from 2942 base pairs to 66,739 base pairs (Table S1). These results suggest that there are two tandemly duplicated gene clusters (TDGCs) of the *DFR* gene in the *D. cambodiana* genome; however, further research is required to verify the findings. We temporarily named the *DFR* gene cluster on chromosome 5 as TDGCs-1 (*DcDFR1*–*5*) and the *DFR* gene cluster on chromosome 2 as TDGCs-2 (*DcDFR9*–*12*, *16*).

To comprehensively understand the evolutionary progression of the *DcDFR* gene family in *D. cambodiana*, an in-depth investigation into gene duplication events was conducted. The investigation included both within *D. cambodiana* itself, and between *D. cambodiana* and the model plants *O. sativa* and *A. thaliana*. A total of five pairs of collinear genes were identified in *D. cambodiana* through intraspecific collinearity analysis (Fig. 1D). These pairs were located on chromosomes 2, 6, 10, and 19. The identified gene pairs were *DcDFR8*/*DcDFR14*, *DcDFR8*/*DcDFR17*, and *DcDFR14*/*DcDFR17*, as well as two additional gene pairs involving *DcDFR7*, *DcDFR10*. The presence of five pairs of collinear genes indicates that the *DFR* gene family of *D. cambodiana* has undergone multiple gene duplication events. However, no collinear genes were found within TDGCs-1 or TDGCs-2.

The interspecific collinearity analysis detected 16 collinear gene pairs between *D. cambodiana* and *O. sativa*. In contrast, only three collinear gene pairs were identified between *D. cambodiana* and *A. thaliana* (Fig. 1E and Table S2). The high collinearity of the *DFR* gene family observed between *D. cambodiana* and *O. sativa* indicates that the gene family has a high degree of conservation among monocotyledonous plants, while the significant differences observed between *D. cambodiana* and *A. thaliana* indicate variations in the gene family differentiation pathways between monocotyledonous and dicotyledonous plants.

### Gene structure and conserved domains of *DcDFR* gene family

**Fig. 2:**
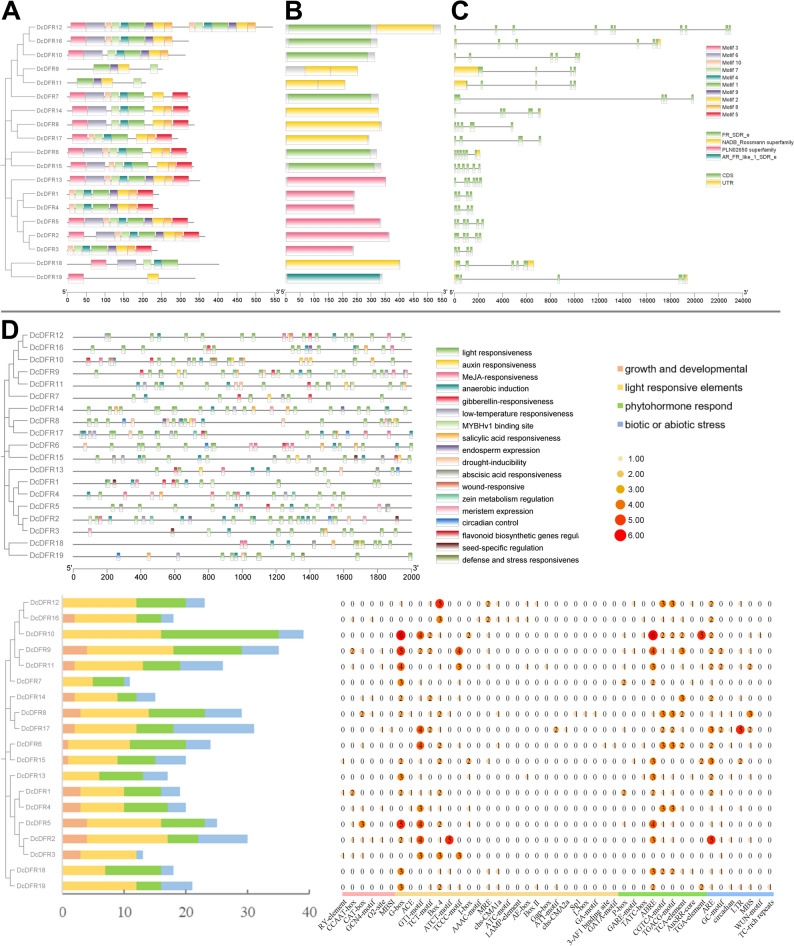
Comprehensive structural and functional characterization of the DFR gene family in *D. cambodiana*. **A-B** The conserved motif and domain of the *DcDFR* gene family. **C** The intron–exon structure of the *DcDFR* genes. **D** Predicted structures of *cis*-acting elements in the promoter region of the *DcDFR* gene family

An analysis was conducted on the genetic structure and conserved motifs of the *DcDFR* gene family using the *D. cambodiana* genome database (Fig. 2A, B and C). MEME software was used to predict 10 distinct motifs and TBtools was used to analyze conservation of the *DcDFR* proteins. The lengths of the conserved motifs ranged from 15 to 50 amino acids (Table 3) and were unevenly distributed among the DcDFR proteins (Fig. 2A). Notably, motif 3 was predominantly located at the N-terminus of most proteins. The most prevalent motifs among the 19 DcDFR proteins were motifs 1 (18 DcDFRs), 2 (18 DcDFRs), 7 (18 DcDFRs) followed by motifs 4 (16 DcDFRs), 3 (14 DcDFRs), 8 (14 DcDFRs), 5 (12 DcDFRs), and 6 (12 DcDFRs). Motif 9 was uncommon (11 DcDFRs). DcDFR12 had the highest number of motifs (16), whereas DcDFR19 had the lowest number of motifs (2). Generally, the presence of diverse conserved motifs could potentially contribute to the functional differentiation among DcDFR proteins (Fig. 2B). The PLN02650 superfamily domain comprised eight motifs, namely motifs 1, 2, 4, 5, 7, 8, 9, and 10. Alterations, such as replacement or loss of these motifs, could lead to changes in the protein function. The FR_SDR_e domain and AR_FR_like_1_SDR_e domain, both belonging to the Short-chain Dehydrogenase/Reductase (SDR) superfamily, contained motifs 2 and 3. The results suggest that these two motifs are highly conserved within the SDR superfamily and are crucial for maintaining the structural and functional integrity of proteins within this family.

**Table 3:**
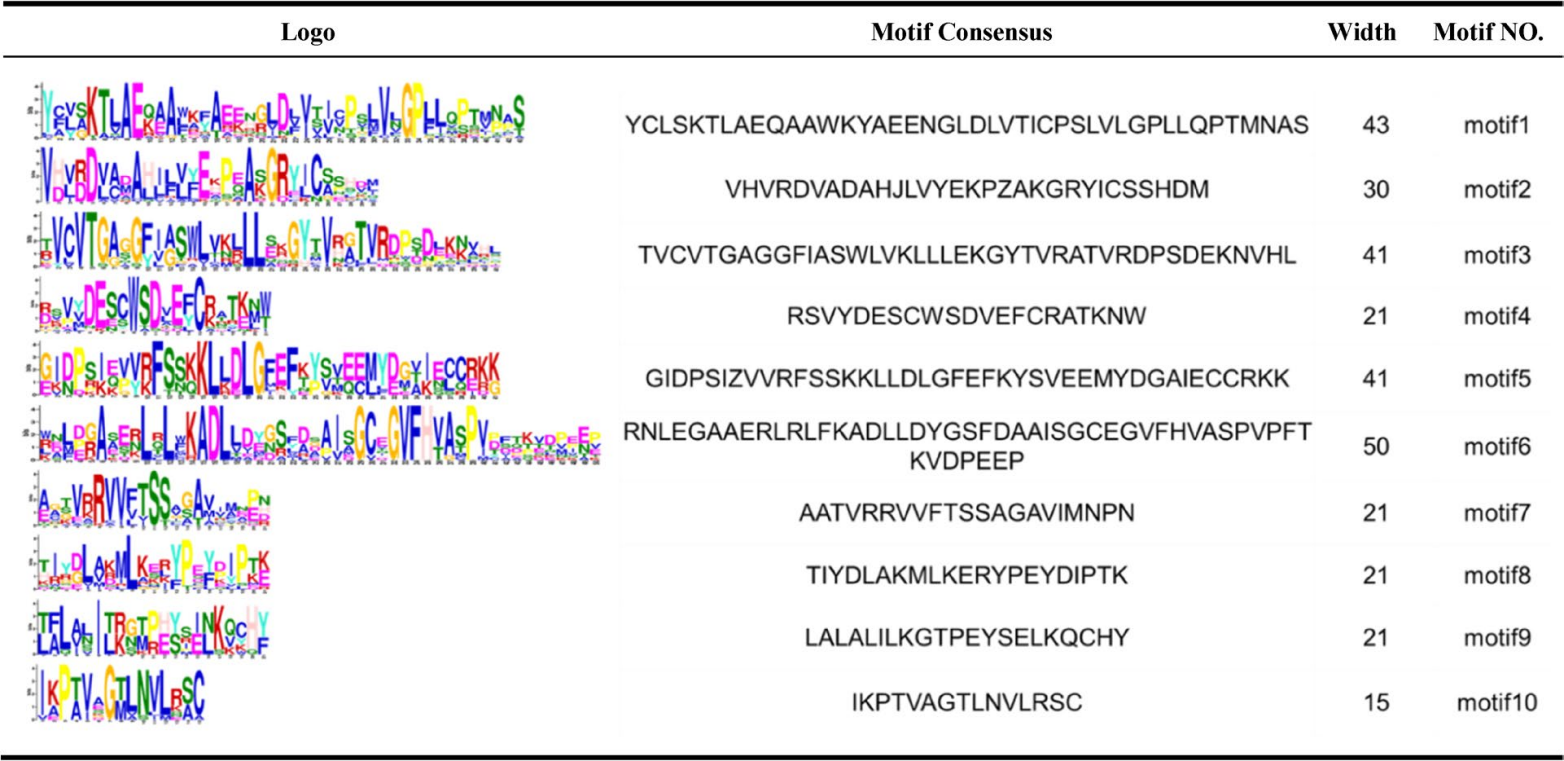
Specific conserved motifs of the DcDFR gene family in *D. cambodiana*

Analysis of the structural characteristics of these genes (Fig. 2C) revealed that over 94% (18 out of 19 *DcDFRs*) of the gene family members contained 3–5 introns and 4–6 exons. The finding indicates that the vast majority of *DcDFR* genes follow the specific intron–exon combination pattern during splicing. Notably, *DcDFR12* deviated from the typical pattern by adopting an alternative splicing mechanism and possessing the highest number of introns (9) and exons (10) within the *DcDFR* gene family.The differences observed in protein motifs, domains, and gene structures indicate that the *DFR* gene family of *D. cambodiana* has certain characteristics of evolutionary complexity and functional diversity. Remarkably, five genes within the TDGCs-1 gene cluster were relatively similar in terms of motifs, domains, and gene structures; however, significant differences were observed among genes in the TDGCs-2 cluster.

### Prediction of cis-acting elements of *DcDFR* gene promoter

*Cis*-acting elements, which are transcription factor binding sites, play a pivotal role in regulating the initiation of gene transcription. Various *cis*-acting elements were involved in responses to light, phytohormones, abiotic stress, and plant development (Fig. 2D). A total of 23 *cis*-acting elements that are functionally associated with light responsiveness were identified (Fig. 2D and Table S3). Specifically, each *DcDFR* gene contained 5–16 light-responsive elements, including the G-box, Box 4, TCCC-motif, I-box, and GT1-motif, which are known for their roles in light-related transcriptional regulation [55]. Six *cis*-acting elements were associated with the plant development process. These elements were involved in seed-specific regulation, meristem expression, endosperm expression, zein metabolism regulation, flavonoid biosynthesis gene regulation, and MYBHv1 binding site. The flavonoid biosynthetic gene regulation element was only detected in *DcDFR17* (Table S3). Nine *cis*-acting elements were related to phytohormones, such as elements responsive to gibberellin, abscisic acid, methyl jasmonate, salicylic acid, and auxin. All members of the *DcDFR* gene family shared this category of phytohormone-related elements, except *DcDFR3*. Furthermore, all promoters of *DcDFR* genes contained *cis*-acting elements involved in abiotic stress response regulation. These *cis*-acting elements included anaerobic induction, low-temperature responsive, drought-inducibility, wound-responsive, circadian control, as well as defense- and stress-responsive *cis*-elements. However, the wound-responsive and defense and stress responsive gene regulation *cis*-elements were only detected in *DcDFR10* and *DcDFR19*, respectively (Table S3). Dragon’s blood synthesis is closely associated with light, hormone, and stress responses, and secondary metabolic processes during *Dracaena* plant development. Overall, the results of this study showed that the promoter region of the *DFR* gene in *D. cambodiana* contains response elements for these processes, indicating that the *DFR* gene is one of the key factors influencing dragon’s blood synthesis.

### Phylogenetic tree construction and prediction of expression characteristics of *DFR* gene family

**Fig. 3:**
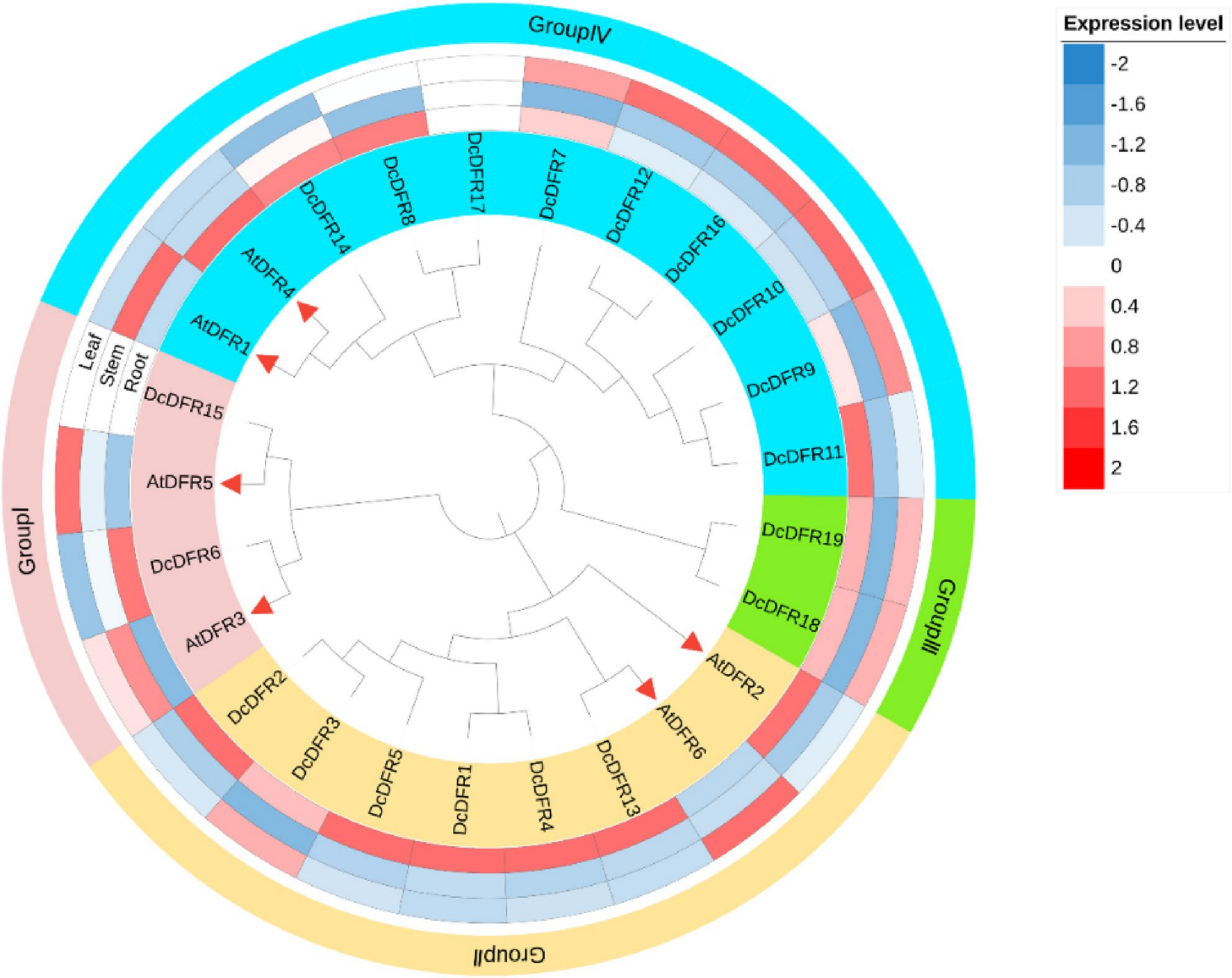
Phylogenetic tree and prediction of the expression characteristics of the *DFR* gene family. Red triangles represent the DFR proteins from *A. thaliana*. *A. thaliana* leaves were collected from the entire rosette after transitioning to flowering and the stems were from the second internode

To elucidate the classification and evolutionary relationships within the *DFR* gene family in *D. cambodiana*, a phylogenetic tree was constructed using 19 proteins (DcDFR1–19) from *D. cambodiana* and 6 proteins (AtDFR1–6) from *A. thaliana* (Table S1). According to the results, 25 DFR proteins from the two species were grouped into four subfamilies (Ⅰ–Ⅳ) with 4, 8, 2, and 11 members (Fig. 3). Three subfamilies (Ⅰ, Ⅲ, and Ⅳ) comprised *DFR* genes from both *D. cambodiana* and *A. thaliana*, while subfamily Ⅱ consisted of only two *DFR* genes. The *DFR* genes within the TDGCs-1 and TDGCs-2 gene clusters were grouped into the same branch, indicating that the genes within these clusters are evolutionarily homologous.

To further explore the functions of the *DFR* gene family, gene expression data of *D. cambodiana* were obtained from the NCBI database and those of *A. thaliana* were obtained from the ePlant website. We selected roots, stems, and leaves to generate a heatmap of gene expression data, which was then combined with the phylogenetic tree (Fig. 3). Gene expression analysis revealed that *DcDFR15* and *DcDFR17* were not expressed in the roots, stems, and leaves of *D. cambodiana*. However, the expression of the remaining 17 *DcDFR* genes was upregulated in either the roots or leaves. The expression levels of nine *DFR* genes (*DcDFR1*, *DcDFR2*, *DcDFR4*, *DcDFR5*, *DcDFR*6, *DcDFR8*, *DcDFR11*, *DcDFR13*, and *DcDFR14*) in *D. cambodiana* root tissues were higher than those in stem and leaf tissues. The expression levels of five *DFR* genes (*DcDFR7*, *DcDFR9*, *DcDFR10*, *DcDFR12*, and *DcDFR16*) in leaf tissues were higher than those in root and stem tissues. Moreover, a substantial variation was observed in the expression patterns of genes belonging to the same subfamily. The results indicate that the expression of most *DFR* genes in *D. cambodiana* has a certain degree of tissue specificity and some of the genes that are sensitive to environmental signals are located in the root and leaf tissues. Genes within the TDGCs-1 and TDGCs-2 clusters were co-expressed, although each had tissue-specific expression. Specifically, TDGCs-1 was co-expressed in roots and TDGCs-2 was co-expressed in leaves.

### Expression of *DcDFR*s in different tissues of *D. cambodiana* A

**Fig. 4:**
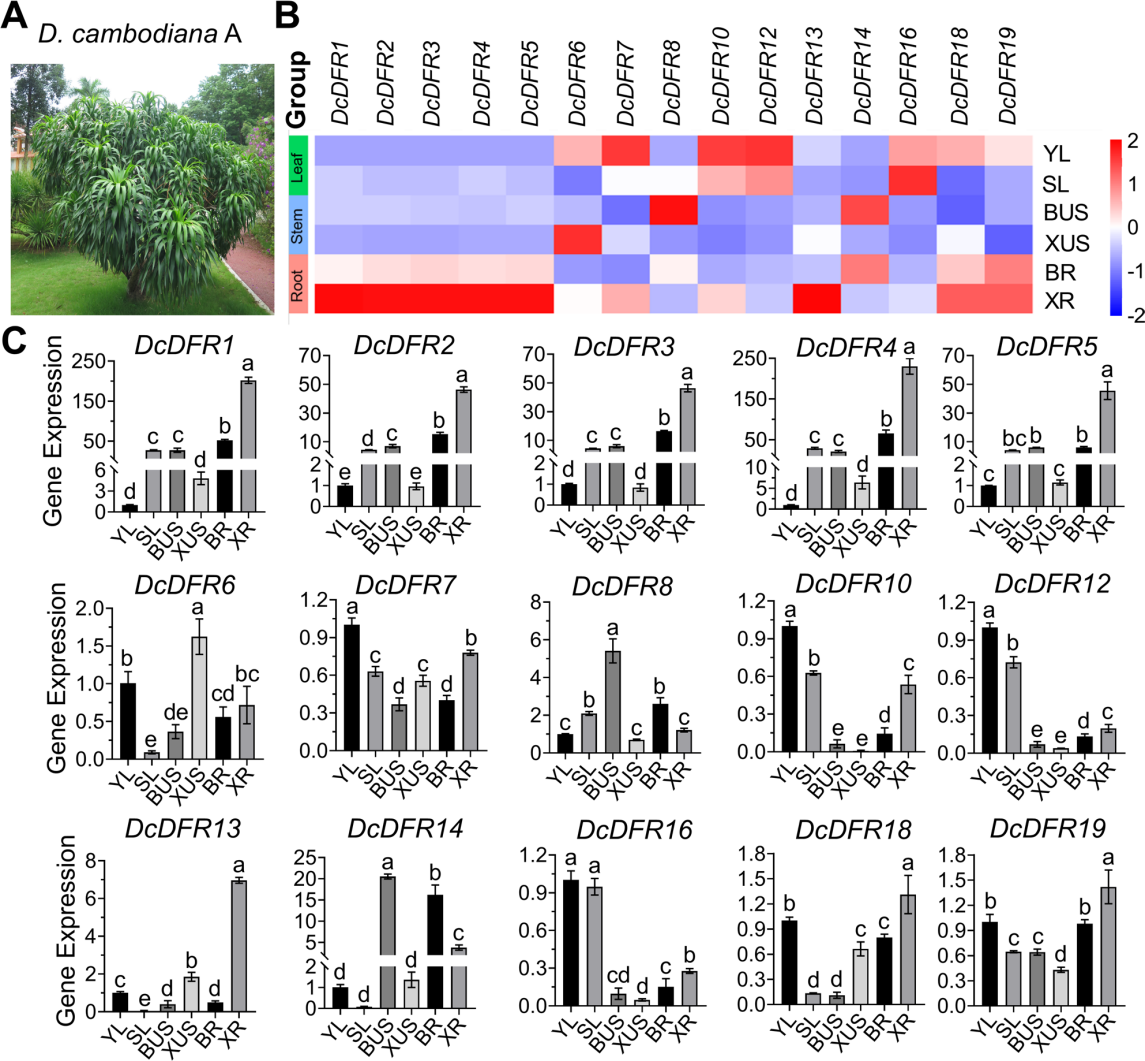
Morphology and gene expression in *D. cambodiana* A tissues. **A** Morphology of *D. cambodiana* A tree. **B** Gene expression levels were normalized according to the column scales and visualized in a heatmap. YL, young leaves; SL, senescent leaves; BUS, bark of the undamaged stems; XUS, xylem of the undamaged stems; BR, bark of the roots; XR, xylem of the roots. **C** The relative expression levels of *DcDFR* genes in different tissues. Error bars indicate the standard deviation of three biological replicates; different letters indicate significant differences at *P* < 0.05 by one-way ANOVA

*D. cambodiana* A represents the Yunnan clade, characterized by soft leaves [1] (Fig. 4A). A previous study conducted by our research team revealed that dragon’s blood accumulates both in the roots and damaged stems of *D. cambodiana* A, and its content in the bark is higher than that in the xylem [56]. Moreover, the study revealed that the expression level of *DFR* was high in roots and damaged stems. In this study, the expression patterns of the *DFR* gene family (Fig. 4B and 4C) were analyzed using the same samples used in the previous study [56]. Taking the expression level of the *DcDFR* gene in young leaves (YL) as a reference, a bar graph that quantitatively depicts the relative gene expression levels across diverse tissues was constructed. The results showed that the expression levels of eight *DFR* gene family members, namely *DcDFR1*, *DcDFR2*, *DcDFR3*, *DcDFR4*, *DcDFR5*, *DcDFR13*, *DcDFR18*, and *DcDFR19* in the root xylem (XR) were high. The expression levels of four *DRF* gene family members, namely *DcDFR7*, *DcDFR10*, *DcDFR12*, and *DcDFR*16 in YL were high. However, the expression levels of *DcDFR9*, *DcDFR11*, *DcDFR15*, and *DcDFR17* were not detected in all samples. The variation in gene expression patterns indicates functional divergence among members of the *DcDFR* gene family, suggesting that *DcDFR1*, *DcDFR2*, *DcDFR3*, *DcDFR4*, *DcDFR5*, *DcDFR13*, *DcDFR18*, and *DcDFR19* could be involved in the biosynthesis of dragon’s blood in *D. cambodiana* A. The qPCR results showed that genes within the TDGCs-1 and TDGCs-2 clusters were co-expressed and tissue-specific, which is consistent with the predicted results. However, *DcDFR9* and *DcDFR11* in the TDGCs-2 cluster were not expressed.

### *DcDFR* expression and flavonoid content in *D. cambodiana* A stem after wound induction

**Fig. 5:**
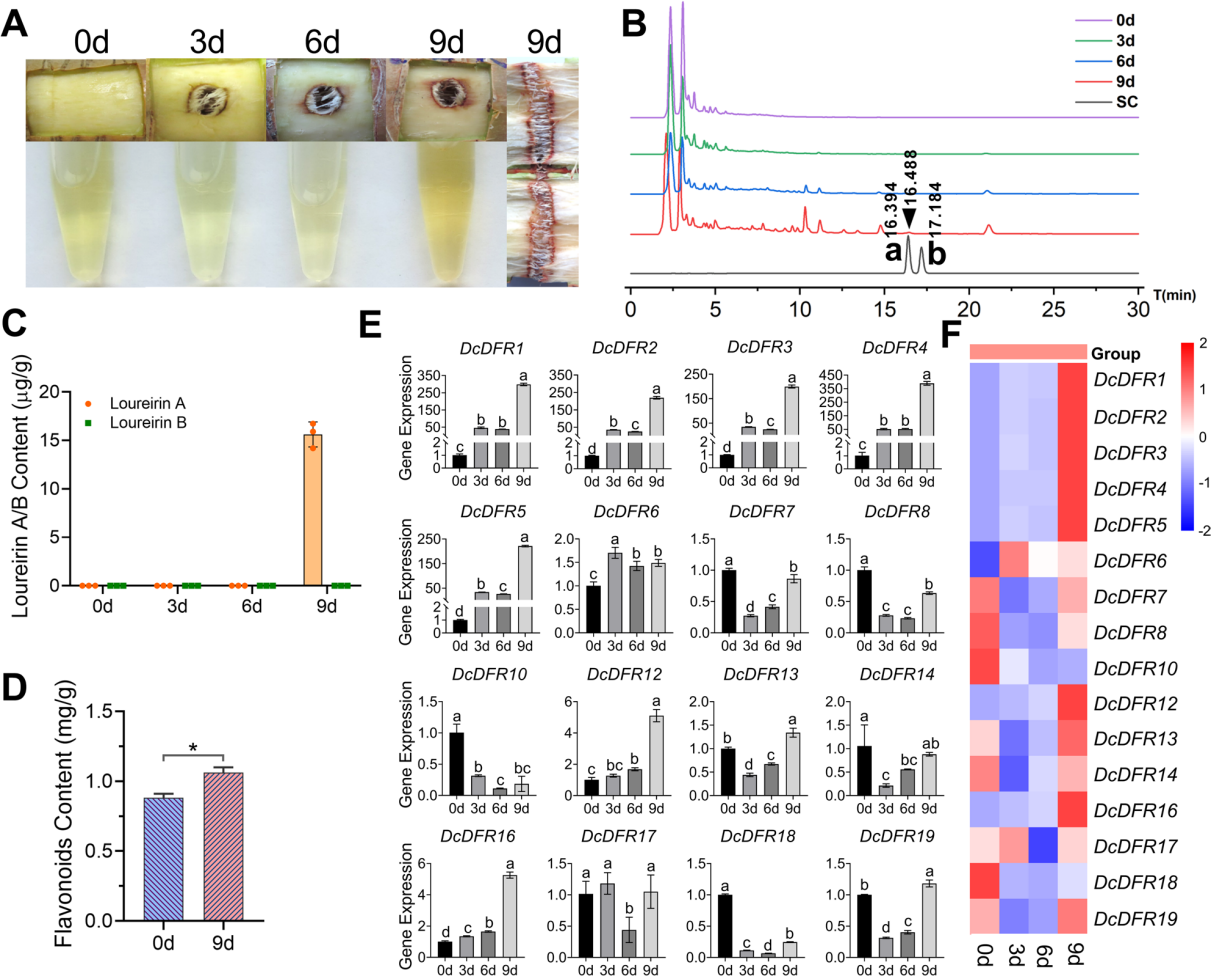
Multidimensional analysis of stem responses to wound induction in *D. cambodiana* A. **A** The changes in stems and methanol extracts from stems. **B**-**C** HPLC analysis of loureirin A/B content in stems after 0, 3, 6, and 9 d of wound induction. SC, standard compounds; “a” and “b” represent loureirin A and B, respectively and the black triangle represents loureirin A peak. The values are means ± standard deviation (SD) of three biological replicates. **D** The flavonoid content of stems after 0 and 9 d of wound induction. Error bars represent the SD of three biological replicates, *P* = 0.0198; * denotes *P* < 0.05 by Student’s test. **E** Variations in the expression levels of *DcDFR* genes in *D. cambodiana* A stems after 0, 3, 6, and 9 d of wound induction. Error bars represent the SD of three biological replicates; different letters indicate significant differences at *P* < 0.05 by one-way ANOVA. **F** Gene expression levels were normalized according to the scale of rows and visualized in a heatmap

To further explore whether the *DFR* gene is involved in the biosynthetic reaction of dragon’s blood, undamaged and healthy *D. cambodiana* A stems were selected for wound induction (Fig. 5A). Wound color gradually intensified and turned brown as the induction time progressed. The stems that had been subjected to wound induction for nine days were longitudinally sectioned and distinct brown substances were observed to have formed around the wound. Similarly, methanol extract color changed from light yellow to dark yellow. High performance liquid chromatography (HPLC) analysis revealed that the contents of compounds in the stem increased substantially after nine days of wound induction and loureirin A was detected in the samples after nine days (Fig. 5B and 5C). The total flavonoid content in samples was determined after 0 and 9 d of wound induction (Fig. 5D). The results showed that the total flavonoid content after 9 d of wound induction was significantly (*P* < 0.05) higher than that after 0 d of wound induction.

The expression levels of all *DcDFR* genes in *D. cambodiana* A following wound induction were analyzed (Fig. 5E and 5F). The results demonstrated that the expression levels of *DcDFR9*, *DcDFR11*, and *DcDFR15* were undetectable. The expression levels of the other *DcDFR* genes varied during the wound induction period (0–9 d). The expression levels of *DcDFR1*,* DcDFR2*, *DcDFR3*, *DcDFR4*, *DcDFR5*, *DcDFR12*, and *DcDFR16* increased with an increase in the induction time and their expression patterns were largely consistent when compared to those of the control samples (0 d). The expression levels of *DcDFR7*, *DcDFR8*, *DcDFR13*, *DcDFR14*, *DcDFR18*, and *DcDFR19* initially decreased and subsequently increased after wounding. These results indicate that *DcDFR1*–*5* could be potential genes involved in resin formation in *D. cambodiana* A after wound induction. Genes within the TDGCs-1 and TDGCs-2 clusters were co-expressed in tree stems under wound stress and their expression levels increased with the prolongation of trauma period. However, the expression of *DcDFR10* in the TDGCs-2 cluster was suppressed under wound stress. *DcDFR9* and *DcDFR11* in the TDGCs-2 cluster were not expressed. In addition, *DcDFR17* was not expressed in various tissues of *D. cambodiana* A, but was expressed under wound stress, indicating that *DcDFR17* could be associated with wound stress resistance.

### Expression of *DcDFR* genes and dragon’s blood content in different tissues of *D. cambodiana* B

**Fig. 6:**
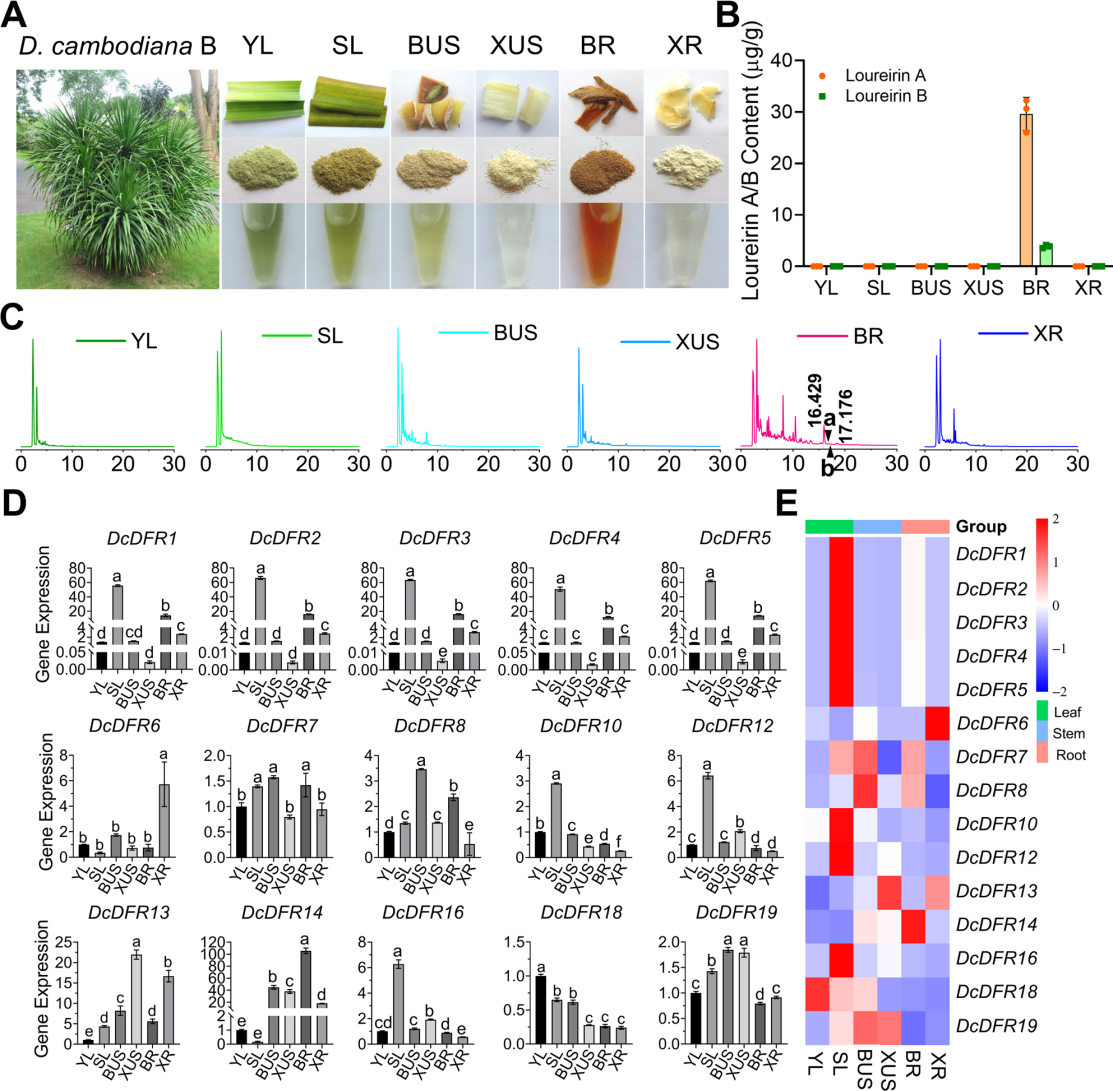
Multidimensional analysis of *D. cambodiana* B tissues. **A** Morphology of *D. cambodiana* B tree and characteristics of different tissues, including the morphology of fresh samples, dried powder samples, and methanol extracts of different tissues. YL, young leaves; SL, senescent leaves; BUS, bark of the undamaged stems; XUS, xylem of the undamaged stems; BR, bark of the roots; XR, xylem of the roots. **B** The contents of loureirin A and B in different tissues. The values are means ± standard deviation (SD) of three biological replicates. **C** Chromatograms of methanol extracts from different *D. cambodiana* B tissues. “a” and “b” at the position of the black triangle represent loureirin A and B, respectively. **D** The relative expression levels of *DcDFR* genes in different *D. cambodiana* B tissues. Error bars represent the SD of three biological replicates; different letters indicate significant differences at *P* < 0.05 by one-way ANOVA. **E** Gene expression levels were normalized according to the scale of rows and visualized in a heatmap

Research has revealed that *D. cambodiana* A and *D. cambodiana* B are two branches of *D. cambodiana *[1]. To gain a more in-depth understanding of the expression patterns of *DcDFR* genes in *D. cambodiana*, *D. cambodiana* B tissue samples were collected (Fig. 6A). The extract from BR exhibited a red color (Fig. 6A). Loureirin A and B were only detected in BR samples and loureirin A content was significantly (*P* < 0.01) higher than that of loureirin B (Fig. 6B). From the chromatogram (Fig. 6C), we observed that flavonoids in the methanol extract from BR were more abundant. A comprehensive analysis of the expression levels of *DcDFR* genes in the samples was performed (Fig. 6D and 6E). The results revealed that the expression patterns of the *DcDFR* gene family in different *D. cambodiana* B tissues varied substantially. The expression levels of *DcDFR9*, *DcDFR11*, *DcDFR15*, and *DcDFR17* were not detected, which was consistent with the observation made in *D. cambodiana *A. However, the expression patterns of the other genes varied between *D. cambodiana* A and *D. cambodiana* B tissues. Eight genes, *DcDFR1*, *DcDFR2*, *DcDFR3*, *DcDFR4*, *DcDFR5*, *DcDFR10*, *DcDFR12*, and *DcDFR16*, had the highest relative expression levels in senescent leaves (SL). *DcDFR1*, *DcDFR2*, *DcDFR3*, *DcDFR4*, and *DcDFR5* exhibited similar expression patterns, with the second-highest expression levels being observed in the bark of roots (BR) and the lowest in the xylem of undamaged stems (XUS). *DcDFR13* and *DcDFR19* exhibited relatively high expression levels in XUS. The expression levels of *DcDFR7* and *DcDFR8* were the highest in the barks of undamaged stems (BUS); *DcDFR6* had the highest expression level in the xylem of roots (XR); *DcDFR18* had the highest expression level in YL; and *DcDFR14* had the highest expression level in the bark of roots (BR). The *DFR* genes within the TDGCs-1 and TDGCs-2 clusters were co-expressed and tissue-specific in *D. cambodiana* B. However, *DcDFR9* and *DcDFR11* within the TDGCs-2 cluster were not expressed in *D. cambodiana* B tissues. Based on the qRT-PCR and HPLC results, dragon’s blood resin contains a large quantity of flavonoids; therefore, we speculated that *DcDFR1*, *DcDFR2*, *DcDFR3*, *DcDFR4*, *DcDFR5*, *DcDFR7*, *DcDFR8*, and *DcDFR14* genes could be involved in dragon’s blood resin synthesis in *D. cambodiana* B.

### Expression of *DcDFR* genes and flavonoid content in *D. cambodiana* B stems after wound induction

**Fig. 7:**
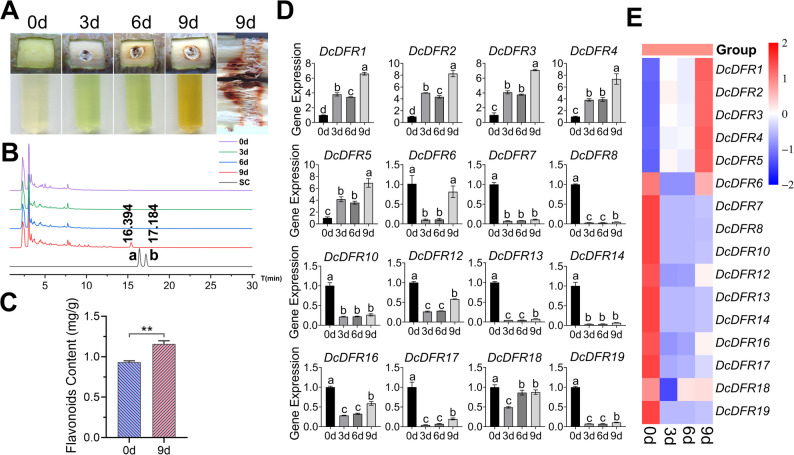
Variations in gene expression and flavonoid content in *D. cambodiana* B stems after wound induction. **A** Variations in the colors of stems and methanol extracts from stems. **B** HPLC analysis results of methanol extracts from stems 0, 3, 6, and 9 d after wound induction. SC, standard compounds; “a” and “b” represent loureirin A and B, respectively. **C** Flavonoid content of *D. cambodiana* stems after 0 and 9 d of wound induction. Error bars represent the standard deviation (SD) of three biological replicates, *P* = 0.0057; ** denote *P* < 0.01 by Student’s test. **D** Variations in *DcDFR* gene expression levels in *D. cambodiana* stems after 0, 3, 6, and 9 d of wound induction. Error bars represent the SD of three biological replicates; different letters indicate significant differences at *P* < 0.05 by one-way ANOVA. **E** Gene expression levels were normalized according to the scale of rows and visualized in a heatmap

The undamaged and healthy stems of *D. cambodiana* B were selected for wound induction (Fig. 7A). The color of the wound and methanol extracts was initially similar to those of *D. cambodiana* A, then gradually intensified, and eventually turned brown as induction time increased. The compound contents increased significantly after 9 d of wound induction (Fig. 7B), but loureirin A and B were not detected in the samples. The results showed that the total flavonoid content at 9 d of wound induction was significantly higher than that at 0 d of wound induction (Fig. 7C).

The analysis results of *DcDFR* gene expression patterns in *D. cambodiana* B following wound induction are presented in Fig. 7D and 7E. The expression levels of three genes, namely *DcDFR9*, *DcDFR11*, and *DcDFR15* were not detected after wound induction. The expression levels of *DcDFR1*, *DcDFR2*, *DcDFR3*, *DcDFR4*, and *DcDFR5* generally increased with an increase in wound induction time and exhibited similar expression patterns, which is consistent with the observation made in *D. cambodiana* A. The expression levels of three genes, namely *DcDFR7*, *DcDFR10*, and *DcDFR14*, decreased significantly after wound induction. The expression levels of *DcDFR6*, *DcDFR8*, *DcDFR12*, *DcDFR13*, *DcDFR16*, *DcDFR17*, *DcDFR18*, and *DcDFR19* initially decreased and then increased after wound induction. These results indicate that *DcDFR1*–*5* genes are potential genes involved in resin formation in *D. cambodiana* B after wound induction. *DFR* genes within the TDGCs-1 and TDGCs-2 clusters were co-expressed in *D. cambodiana* B stems under wounding stress. However, unlike *D. cambodiana* A, the expression of genes within the TDGCs-2 cluster was downregulated.

## Discussion

The *DFR* gene plays a key role in modulating anthocyanin synthesis, plant growth, and development [19]. The gene also enhances substantially improve plant resistance to various biotic and abiotic stresses, such as high temperature, drought, salinity, and cold [57, 58]. To date, the *DFR* gene family has been identified and characterized in numerous plant species, including strawberry [35], rapeseed [59], apple [60], and rice [61]. Nevertheless, a comprehensive and in-depth research on the *DFR* gene family in the *Dracaena* genus has not been conducted.

During the evolutionary course of diverse species, gene families commonly undergo tandem duplication or large-scale segmental duplication events, which directly impact the expansion of gene families [62]. According to a previous study, *D. cochinchinensis*, a close relative of *D. cambodiana*, has experienced two whole-genome duplication (WGD) events [53]. The karyotypes of *D. cambodiana* and *D. cochinchinensis* are 2n = 40 [63, 64]. Therefore, we hypothesized that the WGD event could be a primary driving force behind the expansion and evolution of the *DFR* gene family in *D. cambodiana*. The reference genome of *D. cambodiana* used in this study was a haploid genome (haplotypes A and B). Based on the assembly information, haplotype A database, which had a larger size, a higher assembly rate, and more annotated protein-coding genes, was selected as the reference genome. Five pairs of segmental duplication genes, three pairs of segmental duplication genes among the 19 *DFR* genes, and two gene pairs, *DcDFR7* and *DcDFR10* exhibited collinearity with two genes that have not been confirmed to be *DFR* genes, which were located on chromosomes 19 and 6, respectively (Fig. 1D). A prediction based on the published whole-genome of *D. cochinchinensis* revealed 31,619 protein-coding genes [53] and GeneID predicted that the *D. cambodiana* genome contained 53,700 genes [65]. However, the number of protein-coding genes in the selected haplotype A was fewer than that in the published genome. The finding suggests that using a single haploid genome for gene family research still has certain limitations when compared to conducting research within the whole genome context. Therefore, a further comprehensive screening of the *DFR* gene family within the whole genome context (haplotypes A and B) should be performed to identify new functional *DFR* genes.

The phenomenon of homologous genes with high sequence similarity existing in clusters is extremely common in the plant genome [66], but relatively few gene clusters involved in secondary metabolic pathways have been identified. In this study, a comprehensive investigation of the *DFR* gene family in *D. cambodiana* was conducted. A total of 19 *DFR* genes, which were distributed on six chromosomes, including 2 *DFR* gene clusters, were identified from *D. cambodiana* (Fig. 1C). *DFR* is a key enzyme gene involved in the flavonoid biosynthetic pathway. The common characteristics of secondary metabolic gene clusters in plants are co-expression, co-regulation, and coordinated transcription [67, 68]. In this study, *DcDFR* genes within the TDGCs-1 cluster were located in close proximity on chromosome 5 (Fig. 1C) and they exhibited coordinated transcription and co-expression patterns in different *D. cambodiana* tissues under wounding stress (Figs. 5E and 7D), which demonstrates that they form a gene cluster. The open reading frame sequences of five *DcDFR* genes in the TDGCs-1 cluster were highly homologous to 74.15% (Figure S2 and Table S1) and their intron–exon structures, as well as the conserved motifs and domains of the encoded proteins were extremely similar (Fig. 2A, 2B and 2C). Phylogenetic analysis also showed that they clustered into one clade (Fig. 3). Therefore, TDGCs-1 is highly likely to be a homologous tandemly duplicated gene cluster (TDGCs) formed by tandem duplication events of the *DFR* gene in *D. cambodiana*. In addition, genes in the TDGCs-2 (Locus on chromosome 2 of *D. cambodiana*) cluster exhibited gene cluster characteristics, such as close genomic locations, co-expression, and clustering together in evolutionary analysis. The physical proximity of genes on the genome can enhance co-expression characteristics of gene clusters [68]. Therefore, we hypothesize that it is also a tandemly duplicated gene cluster. Plant gene clusters are generated through gene duplication, neofunctionalization, and dynamic genomic recombination during evolution, and they exhibit the characteristic of independent transcription [67, 69]. Although the five *DFR* genes in the TDGCs-2 cluster showed low sequence similarity, conserved motifs and domains of encoded proteins, and intron–exon structures, two pseudogenes were not expressed, which could be due to gene recombination and neofunctionalization during the gene cluster formation. For example, a TDGCs cluster composed of five O-methyltransferase (*OMT*) genes was identified in the biosynthetic pathway of polymethoxyflavones (PMFs) in citrus, with two of the genes being nonfunctional pseudogenes. Comparative genomic and syntenic analyses have revealed that the *OMT* cluster can be duplicated from *CreOMT6* and it influences the genetic basis of PMF biosynthesis in mandarins through neofunctionalization [54].

TDGCs-2 is a gene cluster composed of three *DFR* genes and two pseudogenes, with the two pseudogenes being derived from the same transcript (Fig. 1C and Table S1). In different tissues of *D. cambodiana* A/B, *DFR* genes within both TDGCs-1 and TDGCs-2 clusters were co-expressed. However, under wounding stress, the TDGCs-2 cluster failed to maintain this co-expression pattern specifically in *D. cambodiana* A (Figs. 5E and 7D). This phenomenon deviates from the typical characteristic of co-expressed gene clusters, indicating functional differentiation within the cluster. *Cis*-element analysis revealed that the promoter of *DcDFR10* contains more abundant light-, hormone-, and abiotic stress-responsive elements compared to other members of TDGCs-2, which provides a mechanistic insight. Specifically, under normal conditions, a shared core regulatory module mediates the co-expression of cluster members; whereas under wounding stress, *DcDFR10* integrates additional environmental signals through its expanded *cis*-elements, leading to the decoupling of its expression from that of other cluster members. We hypothesize that following the formation of the TDGCs-2 cluster in the ancestral species of *Dracaena*, functional differentiation of this gene cluster occurred due to divergent positive selection pressures among species within the genus *Dracaena *[70]. The altered expression pattern of *DcDFR10* is not attributed to gene dysfunction but rather represents an evolutionary adaptation. Through the expansion of its *cis*-regulatory landscape, *DcDFR10* has acquired an enhanced capacity to fine-tune its expression in response to specific environmental cues. Under special circumstances, individual genes within the TDGCs-2 cluster may deviate from the typical co-expression pattern [71, 72]. Ultimately, such regulatory plasticity may enhance the plant’s ability to adapt to complex and varying stress conditions. Generally, an incompletely co-regulated gene cluster is a multifunctional biosynthetic gene cluster that contains at least two partially overlapping gene clusters [73, 74, 75]. Therefore, further research is required to determine whether there are other flavonoid biosynthesis genes associated with the TDGCs-2 gene cluster. If they exist, it will be conducive to determine the unique flavonoid biosynthetic pathways involved in defense response in *Dracaena*.

Oxidative burst, a hallmark early plant defense response, rapidly induces substantial accumulation of reactive oxygen species (ROS) following wound stress. During dragon’s blood formation in *D. cochinchinensis*, ROS functions as a central signaling molecule that activates downstream transcription factors, upregulates the flavonoid biosynthetic pathway, and promotes flavonoid accumulation [53]. The *DcDFR17* gene exhibits a strict wound-specific expression pattern, with no expression detected in healthy tissues. Its promoter region contains three antioxidant response elements (AREs) and one flavonoid biosynthetic regulatory element (MBSI) (Fig. 2D and Table S2). AREs are recognized by transcription factors activated under oxidative stress, indicating that *DcDFR17* expression is likely regulated by wound-induced ROS signaling. Concurrently, the MBSI element may participate in modulating flavonoid metabolism. We hypothesize that this gene not only contributes to flavonoid-mediated defense responses and dragon’s blood synthesis but also aids in maintaining redox homeostasis. Injury-induced ROS signals activate *DcDFR17* expression via these cis-regulatory elements, thereby promoting flavonoid accumulation and enhancing the plant’s pathogen defense and ROS scavenging capacities. Thus, *DcDFR17* may serve as a key integrator of stress signals and metabolic regulation, specifically participating in the formation of dragon’s blood in *D. cambodiana*. Dragon’s blood predominantly comprises flavonoids, which are a highly conserved class of compounds derived from the 2-phenylchromone nucleus [20]. Loureirin A, B, C, and D belong to the chalcone family and represent the primary active constituents, as well as indicator compounds for the quality control of dragon’s blood [76, 77]. According to a previous study, during the induction of dragon’s blood, the synthesis timings of loureirin A, C, and D precede that of loureirin B and the distribution patterns of flavonoid compounds vary at different time points following wounding stress [52]. Chalcones and flavones are mainly enriched in the short term and early to middle phases (within10 d) post wounding, whereas flavonols and isoflavones are predominantly concentrated in the later part of the middle period [52]. This implies that the genes implicated in flavonoid biosynthesis may also exhibit temporal disparities. In this study, the expression levels of five genes *DcDFR1*–*5* (TDGCs-1) in *D. cambodiana* A and B were initially substantially upregulated and then remained unchanged until 9 d after wound stress induction. Conversely, the expression levels of most of the other *DcDFR* genes decreased considerably 3 d after wound stress, with some showing signs of recovery at 9 d after wound stress induction. This phenomenon could be attributed to functional divergence among the 19 *DFR* genes. As the stress duration increased, the genes involved in the middle and later stages of flavonoid biosynthesis restored their catalytic activity. *DcDFR1*–*5* (TDGCs-1) could be involved in the specific flavonoid biosynthetic pathway in *Dracaena* species under wound stress and the final metabolites are associated with the defensive responses of *Dracaena* to wound stress. In plants, gene clusters formed by biosynthetic genes are typically involved only in the metabolic pathway of one or a class of compounds, rather than broader metabolic networks [78]. Loureirin A, B, C, and D are specific plant metabolites in the genus *Dracaena*. However, further studies should be conducted to determine whether the TDGCs-1 and TDGCs-2 gene clusters in *D. cambodiana* are involved in the metabolism of loureirin A, B, C, and D. 

In summary, 19*DFR*genes were identified from the reference genome of*D. cambodiana*and the genes were distributed on six chromosomes, including two tandemly duplicated gene clusters, TDGCs-1 and TDGCs-2. The TDGCs-1 cluster exhibited varying co-expression patterns in different *D. cambodiana*A and B tissues andstems after wound stress, suggesting its involvement in the special flavonoid metabolism and defense response in *D. cambodiana*exposed to wound stress. However, genes within the TDGCs-2 cluster did not follow the typical co-expression pattern when*D. cambodiana*A was subjected to wound stress, which could be caused by functional divergence of the gene cluster under positive selection pressure.

## Conclusion

In this study, 19 *DFR* genes were identified from the reference genome of *D. cambodiana* and the genes were distributed on six chromosomes, including two tandemly duplicated gene clusters, TDGCs-1 and TDGCs-2. The TDGCs-1 cluster exhibited varying co-expression patterns in different *D. cambodiana* A and B tissues and stems after wound stress, suggesting its involvement in the special flavonoid metabolism and defense response in *D. cambodiana* exposed to wound stress. However, genes within the TDGCs-2 cluster did not follow the typical co-expression pattern when *D. cambodiana* A was subjected to wound stress, which could be caused by functional divergence of the gene cluster under positive selection pressure.

## Materials and methods

### Plant materials

The plant materials used in this study were sourced from six healthy 30-year-old trees, including three *D. cambodiana* A trees (Fig. 4A) and three *D. cambodiana* B trees (Fig. 6A) that were both cultivated at the Yunnan Branch of the Institute of Medicinal Plant Development, Chinese Academy of Medical Sciences and Peking Union Medical College, Jinghong (22.0058°, 100.7885°), Yunnan, China, and all plants were formally identified by Professor Haitao Li. Roots, stems, and leaves were collected from the two types of trees [56]. For the wound induction experiment, holes were punched on the stems of trees. Each hole measured approximately 5 mm × 5 mm and the spacing between two adjacent holes was approximately 5 cm. Samples were collected at 0, 3, 6, and 9 d after wound induction. All samples were collected with three biological replicates. Subsequently, the samples were immediately frozen in liquid nitrogen and stored at -80℃ to preserve their integrity for subsequent analysis.

### Data sources

The protein sequences of *A. thaliana *[35] and *O. sativa *[61] were downloaded from TAIR (http://www.arabidopsis.org/) and NCBI database (https://www.ncbi.nlm.nih.gov/), respectively. The associated genome assembly and gene annotation files were downloaded from Ensembl Plants website (https://plants.ensembl.org/, accessed on 24 February, 2025). Genome data, annotation files, and RNA-Seq data of *D. cambodiana* were obtained from Genome Warehouse in the National Genomics Data Center (NGDC), Beijing Institute of Genomics, Chinese Academy of Sciences/China National Center for Bioinformation (https://ngdc.cncb.ac.cn/), under the BioProject accession number PRJCA021348 [64].

### Identification and characterization of *DFR* gene family in *D. cambodiana*

We used the AtDFR and OsDFR protein sequences to screen the *D. cambodiana* database using BLASTP (blast-2.15.0+) [79]. The screening led to the identification of 34 DFR proteins (Fig. 1A). Thereafter, TBtools (v2.302) was used to extract the sequences of the 34 proteins [80]. A further screening of these proteins was based on the annotation files and the functional domain (FR_SDR_e) available on the Conserved Domain Database (CDD) of the NCBI website (https://www.ncbi.nlm.nih.gov/cdd/, accessed on 16 January, 2025) [81], as well as the conserved protein-family domain (Pfam_pf01370) from the Pfam website (https://pfam.xfam.org/, accessed on 10 January, 2025) [82]. Ultimately, a total of 19 *DFR* genes of *D. cambodiana* were successfully identified. The ExPASy ProtParam online tool (https://web.expasy.org/protparam/, accessed on 21 February, 2025) was used to analyze the amino acid length, molecular weight, theoretical isoelectric point, aliphatic index, and grand average of hydrophobicity of DcDFR proteins [83]. The secondary structural features of the DcDFR protein family were analyzed using the SOPMA algorithm (http://npsa-pbil.ibcp.fr/cgi-bin/npsa_automat.pl?page=/NPSA/npsa_sopma.html, accessed on 24 February, 2025) [84], and the 3D structure models of the DcDFR proteins were predicted using SWISS-MODEL (https://swissmodel.expasy.org/, accessed on 11 February, 2025) [85].

### Subcellular localization, chromosome localization, and collinearity analysis

WoLF PSORT (https://wolfpsort.hgc.jp/, accessed on 28 February, 2025) was used to predict the subcellular localization of DcDFR proteins [86]. Gene Location Visualize from GTF/FFF function in TBtools was used to map the distribution of genes on chromosomes. DNA and annotation files of *A. thaliana*, *O. sativa*, and *D. cambodiana* were downloaded. Interspecies and intraspecies collinearity were generated using One Step MCScanX and Advanced Circos functions integrated in TBtools.

### Analysis of gene structure, conserved motifs, and conserved domains of *DcDFR* gene family

MEME (http://meme-suite.org/, accessed 8 March, 2025) was used to analyze the conserved motifs of DcDFR proteins, with the number of motifs being set to 10 [87]. The CDD of the NCBI (https://www.ncbi.nlm.nih.gov/cdd/, accessed on 23 February, 2025) was used to analyze conserved domains. Gene structure, conserved motifs, and conserved domains of *DcDFR* genes were visualized using Gene Structure View (Advanced) function in TBtools.

### Analysis of Cis-acting elements in *DcDFR* gene promoter region

The Gtf/Gff3 Sequence Extract function in TBtools was used to extract the 2000-bp upstream region of the coding sequences (CDs) as the gene promoter region. The promoter sequences were submitted to the PlantCARE database (http://bioinformatics.psb.ugent.be/webtools/plantcare/html/, accessed 4 March, 2025) for the analysis of *cis*-acting elements within the promoter region. The resulting data were visualized using the Simple BioSequence Viewer function in TBtools.

### Evolutionary analysis and tissue-specific expression of *DcDFR* gene family

MEGA-X was used to construct a phylogenetic tree for the *DFR* genes in *A. thaliana* and *D. cambodiana *[88]. The full-length sequences of DFR proteins were aligned using ClustalW and a phylogenetic tree was constructed using the neighbor-joining (NJ) method with a bootstrap value set at 1000. Gene expression data for *A. thaliana* and *D. cambodiana* were downloaded from the Ensembl Plants (http://plants.ensembl.org/index.html, *accessed on 24 February*,* 2025*) and NGDC (https://ngdc.cncb.ac.cn/, accessed on 23 February, 2025) databases, respectively. Gene expression data for three tissues, including root, stem, and leaf tissues were imported into iTOL (https://itol.embl.de/, accessed 12 March, 2025) and combined with a phylogenetic tree. Afterward, iTOL was used to beautify the phylogenetic tree [89].

### RNA extraction and preparation of cDNA test samples

Total RNA was extracted from plant samples using an RNAprep Pure Plant Plus Kit (DP441, Tiangen Biotech, Beijing, China), according to the manufacturer’s instructions. The quality of the extracted RNA was assessed using 1.5% agarose gel electrophoresis and its concentration was determined using a micro-spectrophotometer (NanoDrop One, Thermo Fisher Scientific, USA). The cDNA library was constructed using high quality RNAs and PrimeScript FAST RT Reagent Kit with gDNA Eraser (RR092A, TaKaRa Bio Inc., Shiga, Japan).

### Quantitative RT-PCR (qRT-PCR) analysis

Gene-specific primers (Table S4) for qRT-PCR were designed using Primer Premier 6.0 (PREMIER Biosoft International, Palo Alto, CA, USA) and the actin gene was used as the reference gene. qRT-PCR was performed on a CFX96 Touch Real-Time PCR Detection System (Bio-Rad Laboratories Inc., Hercules, CA, USA) using TB Green Premix Ex Taq II (RR820A, TaKaRa Bio Inc., Shiga, Japan). The PCR program was set as follows: initial denaturation at 95 °C for 30 s, followed by 40 cycles of 5 s at 95 °C for denaturation, and 30 s at 60 °C for annealing and extension. Gene expression levels were calculated using the 2^−∆∆Ct^ method. GraphPad Prism 8.0 (GraphPad Software Inc., La Jolla, CA, USA) was used to generate graphs.

### Extraction of flavonoids

Fresh samples were dried at 60 °C overnight, ground into powder, and sieved through a 60-mesh sieve. Thereafter, 0.1 g of each sample was weighed, 15 mL of methanol was added, and ultrasonic extraction was performed at 25 °C for 15 min. The extraction process was repeated three times. Subsequently, the solvent extracts were combined and methanol was removed using a rotary evaporator at 45°C [90]. The residue was eluted with methanol and the volume was adjusted to 2 mL. The solution was then passed through a 0.22 μm filter membrane in preparation for loureirin A and B, and total flavonoid analysis.

### Loureirin A and B content analysis

HPLC was used to analyze Loureirin A and B contents. HPLC was performed at 25 °C using a Shimadzu LC-2030 C System (Shimadzu, Kyoto, Japan) coupled with a ZORBAX SB-C18 column (5 μm, 4.6 mm × 250 mm; Agilent Technologies, Santa Clara, CA, USA) and a DAD detector. The mobile phase consisted of a gradient of 30% acetonitrile–0.3% acetic acid in water (A) and acetonitrile (B). The initial composition was 100% (A), which changed to 80% (A) at 5 min and was held at this ratio until after 30 min. The flow rate was set at 1.0 mL/min, the injection volume was 10 µL, and the compounds were detected at a wavelength of 278 nm [90]. The standard compounds, loureirin A and B, were obtained from the National Institute for Food and Drug Control (Beijing, China). The concentration/absorbance linear regression equations for loureirin A and B were y=38276x-8635.7 (R^2^ = 0.9999, 0.05–100 µg/mL) and y=24312x + 131.67 (R^2^ = 0.9999, 0.08–10.0 µg/mL), respectively. The contents of loureirin A and B were calculated and expressed as µg per gram of dry sample (µg/g). Origin 2024 (OriginLab Corp., Northampton, MA, USA) was used to plot chromatograms.

### Total flavonoid content analysis

The total flavonoid content was determined using the methods described in Pharmacopoeia of the People’s Republic of China [91]. Briefly, 1 mL of the methanol extract was transferred into a test tube and double-distilled water was added to make the volume up to 5 mL. Thereafter, 0.3 mL of 5% sodium nitrite solution was added to the test tube, the solution was mixed thoroughly, and left to stand for 6 min. Afterward, 0.3 mL of 10% aluminum nitrate solution was added, the solution was shaken thoroughly, and 4.4 mL of 4% sodium hydroxide solution was added after 6 min. The solution was left to stand for 15 min and absorbance was measured at a wavelength of 510 nm. As a standard compound, rutin was obtained from the National Institute for Food and Drug Control (Beijing, China) and the concentration/absorbance linear regression equation was y = 0.0114x + 0.0031 (R^2^ = 0.9995, 5–30 µg/mL). The total flavonoid content was calculated and expressed as mg per gram of dry sample (mg/g). GraphPad Prism 8.0 (GraphPad Software Inc., La Jolla, CA, USA) was used to generate graphs.

### Identification of tandemly duplicated genes clusters

Tandemly duplicated genes clusters (TDGCs) or clusters of tandemly duplicated genes have traditionally been identified subjectively as genomic neighborhoods containing several gene duplicates in close proximity [92]. Generally, tandemly duplicated gene clusters contain homologous genes [54]. In this study, we defined TDGCs as homologous genes located on the same chromosome with an intervening distance of no more than 10-kb between adjacent genes.

### Statistical analysis

All data are presented as means ± standard deviation (SD) and the results are derived from three independent replicates. Unpaired Student’s *t*-test was performed to compare two groups and analysis of variance was performed to compare several groups. All statistical analyses were performed using IBM SPSS Statistics 22.0 (IBM Corp., Armonk, NY, USA). Statistical significance was set at *P < 0.05, **P < 0.01.

## Supplementary Information

Supplementary Material 1.

Supplementary Material 2. Table S1: *DFR* gene family information of *Arabidopsis thaliana*,* Oryza sativa* and *Dracaena cambodiana*, related to Figure 1.

Supplementary Material 3. Table S2: Collinear pairs genes of D. *cambodiana* with *O. sativa* or *A. thaliana*, related to Figure 1E.

Supplementary Material 4. Table S3: Predicted cis-acting element structures in promoter region of *DcDFR* gene family, related to Figure 2D.

Supplementary Material 5. Table S4. Primer sequences of qRT-PCR, related to Figure 4–7.

## Data Availability

The data presented in this study are available on request from the corresponding author.
