# Supplementary material for: Genome-wide identification of the DFR gene family in Dracaena cambodiana and its expression analysis under wound stress
Source: BMC Plant Biol. 2026 Mar 6;26:670. doi: 10.1186/s12870-026-08486-x (PMC13078043; doi:10.1186/s12870-026-08486-x)
Supplement: Supplementary file 1 — Supplementary Material 1. [file 12870_2026_8486_MOESM1_ESM.pdf]

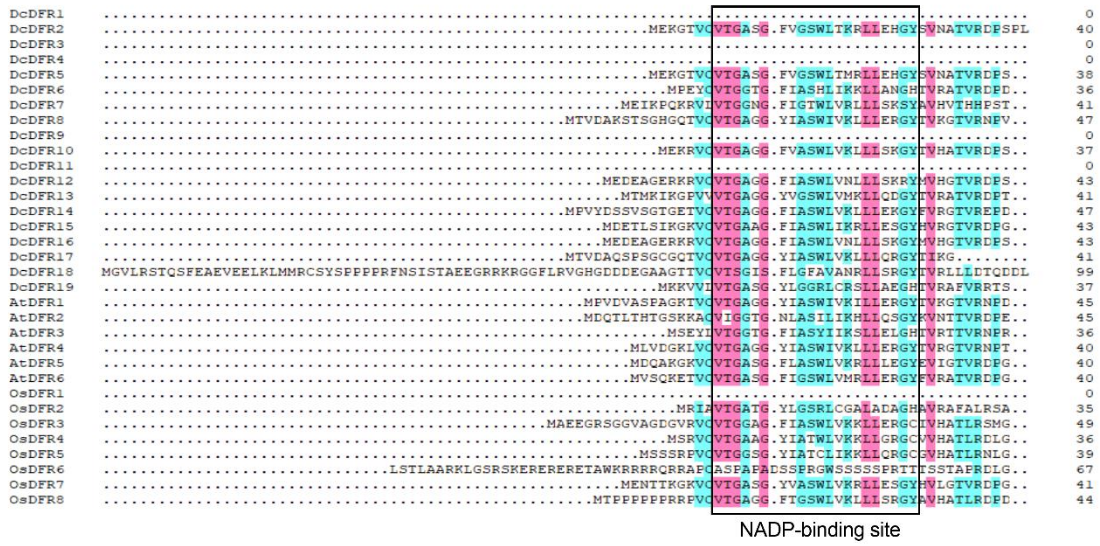

Figure S1 Amino acid sequences alignment of DcDFR with AtDFR and OsDFR protein. The putative NADP-binding site was marked by black box.

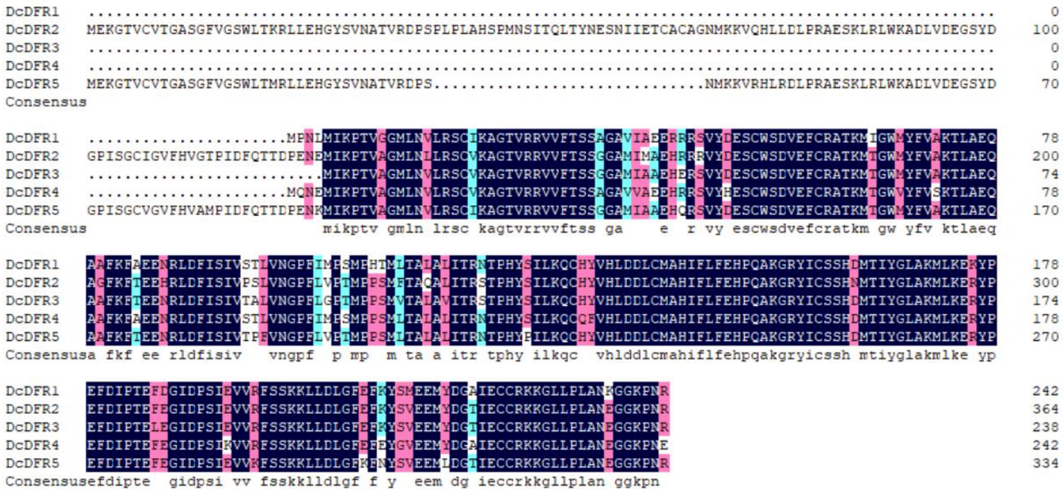

Figure S2 Amino acid sequences alignment of DcDFR1–5.
